# Copper Chelation Therapy Attenuates Periodontitis Inflammation through the Cuproptosis/Autophagy/Lysosome Axis

**DOI:** 10.3390/ijms25115890

**Published:** 2024-05-28

**Authors:** Lujin Zhang, I-Chen Tsai, Zihan Ni, Beichen Chen, Shuaiyuan Zhang, Luhui Cai, Qiong Xu

**Affiliations:** Guanghua School of Stomatology, Guangdong Provincial Key Laboratory of Stomatology, Sun Yat-Sen University, Guangzhou 510055, China; zlujin@163.com (L.Z.); tsaiic@mail2.sysu.edu.cn (I.-C.T.); nizh3@mail2.sysu.edu.cn (Z.N.); chenbch8@mail2.sysu.edu.cn (B.C.); zhangshy227@mail2.sysu.edu.cn (S.Z.); cailh8@mail.sysu.edu.cn (L.C.)

**Keywords:** periodontitis, cuproptosis, copper chelation, mitophagy, lysosomes, macrophages

## Abstract

Periodontitis development arises from the intricate interplay between bacterial biofilms and the host’s immune response, where macrophages serve pivotal roles in defense and tissue homeostasis. Here, we uncover the mitigative effect of copper chelator Tetrathiomolybdate (TTM) on periodontitis through inhibiting cuproptosis, a newly identified form of cell death which is dependent on copper. Our study reveals concurrent cuproptosis and a macrophage marker within murine models. In response to lipopolysaccharide (LPS) stimulation, macrophages exhibit elevated cuproptosis-associated markers, which are mitigated by the administration of TTM. TTM treatment enhances autophagosome expression and mitophagy-related gene expression, countering the LPS-induced inhibition of autophagy flux. TTM also attenuates the LPS-induced fusion of autophagosomes and lysosomes, the degradation of lysosomal acidic environments, lysosomal membrane permeability increase, and cathepsin B secretion. In mice with periodontitis, TTM reduces cuproptosis, enhances autophagy flux, and decreases *Ctsb* levels. Our findings underscore the crucial role of copper-chelating agent TTM in regulating the cuproptosis/mitophagy/lysosome pathway during periodontitis inflammation, suggesting TTM as a promising approach to alleviate macrophage dysfunction. Modulating cuproptosis through TTM treatment holds potential for periodontitis intervention.

## 1. Introduction

Periodontitis manifests as an inflammatory reaction in susceptible individuals who are prone to periodontal microbiota harbored within dental biofilms, leading to the gradual deterioration of the periodontal ligament and alveolar bone [1,2,3]. Globally, more than 65% of people are impacted by periodontitis, with around 11% experiencing its severe variants. Significantly, the occurrence rate rises to 70.1% in adults aged over 65 years old [4,5,6,7]. Results from the 1990–2015 Global Burden of Disease research emphasize the swift increase in worldwide periodontitis cases over ten years, underscoring its rise as a major public health issue and a major hurdle for global health resources [8]. Apart from endangering the health of oral tissues, periodontitis strongly correlates with systemic diseases, including cardiovascular disease, diabetes, and arthritis, greatly impairing the quality of life related to oral health [9,10,11]. The mechanism of systemic diseases aggravated by periodontitis is still ambiguous, but probably includes maladaptive innate immune training, periodontal pathogen bacteremia, and gut microbiota dysbiosis [9,10,11]. Currently, the fundamental approach to managing periodontitis continues to be mechanical therapy [12]. Nonetheless, merely eliminating mechanical plaque is insufficient, highlighting the critical importance of adjusting the host’s immune reaction in treating periodontitis [13].

When pathogenic microbes invade periodontal tissues, a vast array of immune cells, such as macrophages, T cells, B cells, and dendritic cells, are recruited [14]. Macrophages play a crucial role in the destruction of periodontal tissues, where their heightened aggregation and activation lead to tissue lesion [15,16,17,18]. The buildup of inflammatory agents originating from macrophages in gingival tissues and crevicular fluid is linked to the intensity and advancement of periodontitis, while blocking their release is associated with improved bone resorption and the infiltration of inflammatory cells [19,20]. During the advancement of periodontal disease, recruited macrophages experience various forms of cell death, such as pyroptosis, necroptosis, and ferroptosis, leading to inflammatory cytokine sequences that intensify localized inflammation [16,21,22,23]. Significantly, macrophages act as key facilitators in pyroptosis, promoting the release of IL-1β, which, in turn, promotes periodontitis [22]. Furthermore, the occurrence of macrophage necroptosis triggers the release of immunogenic substances within cells, leading to an environment with high proinflammatory activity [21].

The newly identified form of copper-dependent cell death, cuproptosis, results from an overaccumulation of copper that attaches to the lipoylated elements of the mitochondrial tricarboxylic acid cycle, leading to toxic stress and cell death [24,25,26]. Copper is an essential metabolic cofactor in standard amounts and the cellular copper homeostasis is sophisticatedly adjusted by exogenous copper chelators and ionophores [27]. The copper chelators, including Tetrathiomolybdate (TTM) and Bathocuproine disulfonate disodium (BCS), can reduce intracellular copper concentration and inhibit cuproptosis through chelating copper ions, while ionophores such as β-alanyl-l-histidine or Elesclomol assist copper ion transport into cells in cytotoxic or non-toxic ways [27,28]. Research conducted by Tsvetkov et al. depicts a correlation between elevated copper levels in hepatocells and raised lipoylated proteins, suggesting a role of copper build-up in the development of cuproptosis in Wilson disease [24,29]. Significantly, increased levels of copper in the bloodstream of older adults trigger the aggregation of lethal reactive oxygen species (ROS) through cuproptosis, triggering and hastening the aging of blood vessels [30]. Furthermore, the advanced glycosylation end products (AGEs) of diabetic cardiomyocytes intensify cuproptosis through the *Atf3*/*Spi1*/*Slc31a1* route, thereby worsening the condition of diabetic myocardial function [31]. Current research highlights the accumulation of copper ions in macrophages and increased copper concentrations in the saliva of individuals with periodontitis [32,33,34,35]. The analysis of Gene Expression Omnibus (GEO) datasets uncovers notable links between genes associated with cuproptosis and periodontitis, highlighting the urgency for the additional clarification and nature of cuproptosis apart from apoptosis and pyroptosis [26,36]. Nevertheless, the link between macrophage cuproptosis and periodontitis remains unexplored in experimental studies.

The research aimed to explore the molecular underpinnings of the inhibition of cuproptosis in periodontitis by TTM using both in vitro and in vivo models, with the goal of clarifying its possible roles in periodontitis development. For simulating the pro-inflammatory environment of periodontal macropahges, RAW264.7 cells and bone marrow-derived macrophages (BMDMs) exposed to lipopolysaccharide (LPS) were employed to investigate the autophagy flux and the role of lysosomal damage in inhibiting cuproptosis. The research indicated that TTM inhibited cuproptosis, alleviated autophagy flux blockage, attenuated lysosomal damage, and reduced *Ctsb* expression in LPS-activated macrophages. To sum up, our research leads the way in exploring the effects of increased copper on macrophage activity and the involvement of copper chelation TTM therapy in the advancement of periodontitis, providing valuable perspectives for innovative treatments aimed at cuproptosis-induced macrophage inflammation.

## 2. Results

### 2.1. The Expression of Cuproptosis-Related Markers Increases in Periodontitis

**Figure 1:**
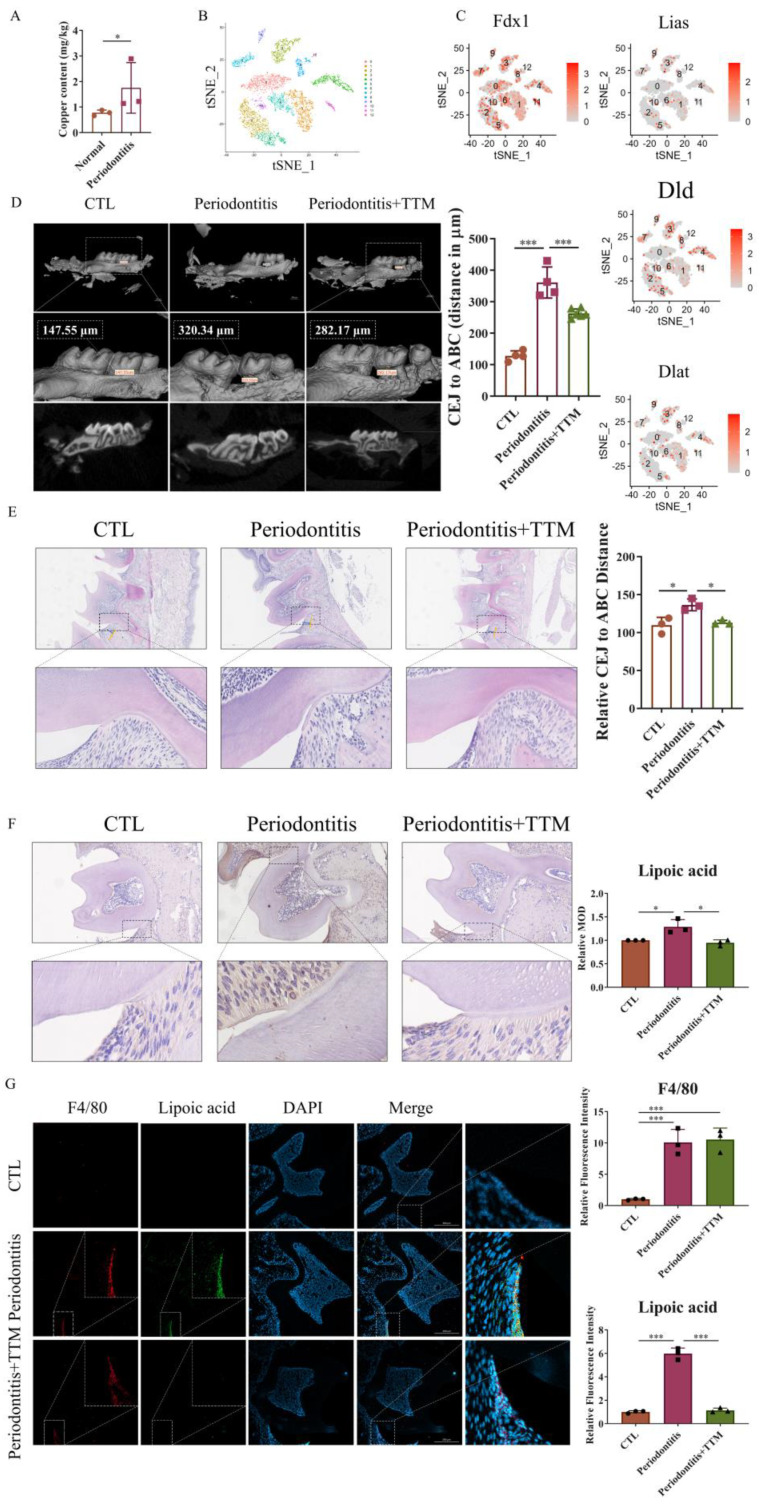
The promotion of cuproptosis-related markers in vivo. (**A**) Inductively coupled plasma mass spectrometer (ICP-MS) of copper in periodontal tissue samples (*n* = 3); (**B**) The t-SNE image of the single-cell profile colored by cluster; (**C**) Feature plots showing expression intensity for cuproptosis-related genes; (**D**) The extent of bone degradation was gauged using the span between the cement–enamel junction (CEJ) and the alveolar bone crest (ABC) (*n* = 3) (scale bar = 500 μm, *n* = 3); (**E**) The HE staining of the gingival tissues sections from each group and quantified by imagej the position of alveolar bone crest is highlighted in yellow and the span between the cement–enamel junction and the alveolar bone crest is highlighted in blue (20×, scale bar = 300 μm, *n* = 3); (**F**) Characteristic images of the immunohistochemical staining of lipoic acid in the periodontal tissue slices (20×, scale bar = 200 μm, *n* = 3); (**G**) Immunofluorescence images showed the co-localization of lipoic acid with F4/80 in periodontal tissues (20×, scale bar = 200 μm, *n* = 3); All results are presented as the mean ± SD. * *p* < 0.05, *** *p* < 0.001.

To investigate the role of cuproptosis in periodontitis, we measured the content of copper ions in periodontal tissues from both periodontitis patients and non- periodontitis individuals. The results revealed elevated copper levels in the periodontal tissues of patients with periodontitis (Figure 1A). Additionally, to assess the expression of cuproptosis-related genes in periodontitis, we analyzed scRNA-seq data (GSE164241) downloaded from the GEO database. Employing t-distributed stochastic neighbor embedding (t-SNE) analysis, we segregated all cells present in the gingival mucosa of periodontitis patients into 11 clusters. Notably, genes associated with cuproptosis, including *Fdx1*, *Lias*, *Dld*, and *Dlat*, were expressed in periodontitis tissue (Figure 1B,C and Figure S1).

To confirm the occurrence of cuproptosis in periodontitis, we established a ligature-induced experimental periodontitis model in mice, with or without the administration of the copper-chelating agent TTM via gavage. Subsequently, we collected both alveolar bones and periodontal tissues for assessment. The data indicated that bone loss was mitigated in the periodontitis+TTM group when compared to the periodontitis group (Figure 1D). Similarly, HE staining revealed a reduction in cavity-like structures in the furcation region of the second molar in the periodontitis+TTM group (Figure 1E). Furthermore, we examined the expression of lipoylation, a representative marker of cuproptosis, in periodontal tissues. The immunohistochemical staining results suggested a significant increase in the lipoylation levels in the periodontitis group, partially reversed as a result of TTM treatment (Figure 1F). Importantly, immunofluorescent analysis revealed the co-localization of the cuproptosis indicator lipoic acid with the macrophage marker F4/80 in the periodontal tissues of the periodontitis group, implying the potential involvement of cuproptosis in macrophages, while TTM inhibited this effect (Figure 1G).

### 2.2. The Expression of Cuproptosis-Related Markers Is Upregulated in LPS-Stimulated Macrophages and Downregulated by TTM Treatment

**Figure 2:**
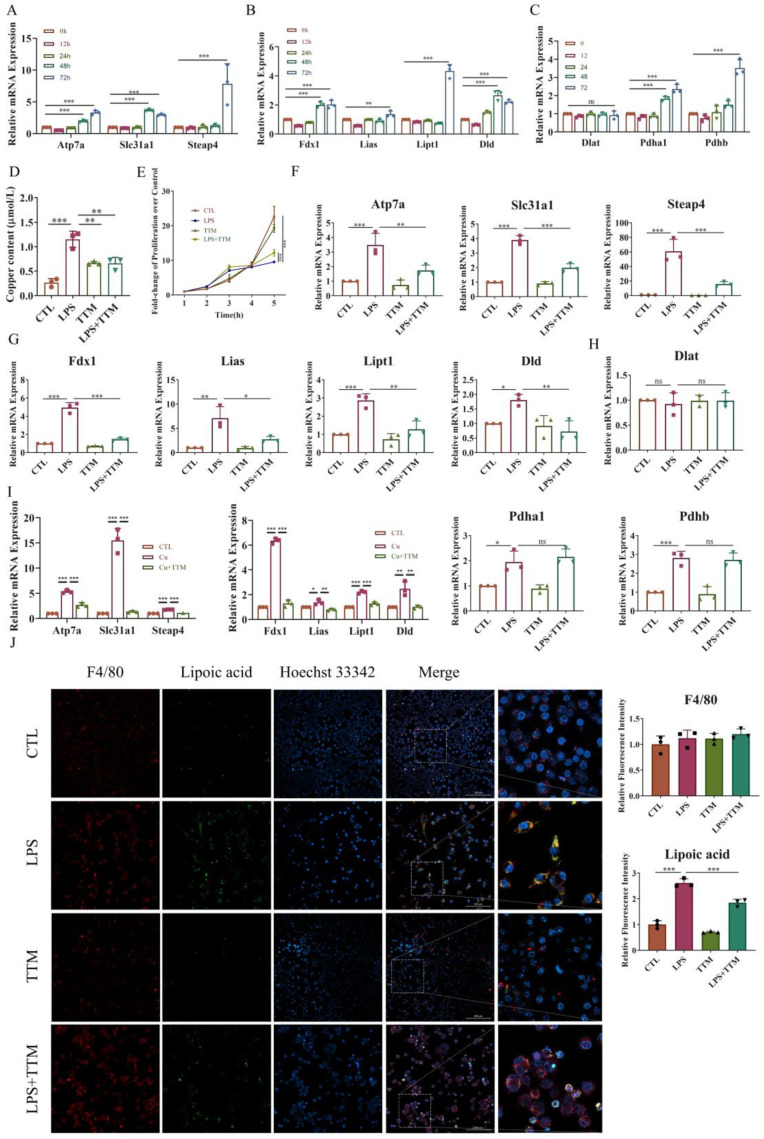
The inhibition of cuproptosis in LPS-stimulated macrophages by TTM. (**A**) The mRNA expression levels of copper transport-related genes (*Atp7a*, *Slc31a1*, and *Steap4*) were quantified using Real-time quantitative reverse transcription-PCR (qRT–PCR) in RAW264.7 cells, and *β-actin* was used as a normalization control (*n* = 3); (**B**) The mRNA expression levels of lipid acid pathway-related genes (*Fdx1*, *Lipt1*, *Lias*, and *Dld*) were quantified using qRT–PCR in RAW264.7 cells, and *β-actin* was used as a normalization control (*n* = 3); (**C**) The mRNA expression levels of lipoylated protein target genes (*Dlat*, *Pdha1*, and *Pdhb*) were quantified using qRT–PCR in RAW264.7 cells, and *β-actin* was used as a normalization control (*n* = 3); (**D**) The copper content of RAW264.7 cells with or without LPS (1 μg/mL) was detected using a cell copper (Cu) colorimetric assay kit (*n* = 3); (**E**) RAW264.7 cells were stimulated with LPS (1 μg/mL) in the presence or absence of TTM (10 μM), and the cell proliferation at 0, 12, 24, 48, and 72 h were measured using the CCK-8 assay (*n* = 3); (**F**) The mRNA expression of copper transport-related genes (*Atp7a*, *Slc31a1*, and *Steap4*) was quantified using qRT–PCR in BMDMs at 72 h, *β-actin* was used as a normalization control (*n* = 3); (**G**) The mRNA expression of lipid acid pathway-related genes (*Fdx1*, *Lipt1*, *Lias*, and *Dld*) was quantified using qRT–PCR in BMDMs at 72 h, *β-actin* was used as a normalization control (*n* = 3); (**H**) The mRNA expression of lipoylated proteins targets genes (*Dlat*, *Pdha1*, and *Pdhb*) was quantified using qRT–PCR in BMDMs at 72 h, *β-actin* was used as a normalization control (*n* = 3); (**I**) qRT-PCR analysis of the mRNA expression of copper transport-related genes (*Atp7a*, *Slc31a1*, and *Steap4*) and lipid acid pathway-related genes (*Fdx1*, *Lipt1*, *Lias*, and *Dld*) in RAW264.7 cells treated with Cu (50 μM) in the presence or absence of TTM (10 μM) for 72 h (*n* = 3); (**J**) Immunofluorescence images showed the co-localization of lipoic acid with F4/80 in RAW264.7 cells at 72 h and quantified using imagej (20×, scale bar = 100 μm, *n* = 3); All results are presented as the mean ± SD. * *p* < 0.05, ** *p* < 0.01, *** *p* < 0.001, and ns for no significant difference.

To investigate the induction of cuproptosis in macrophage inflammation, macrophages were exposed to 1 μg/mL LPS for 0, 12, 24, 48, and 72 h, and the mRNA expression levels of copper transport-related markers (*Atp7a*, *Slc31a1*, and *Steap4*), cuproptosis-related markers (*Fdx1*, *Lipt1*, *Lias*, and *Dld*), and the target genes of lipoylated proteins (*Dlat*, *Pdha1*, and *Pdhb*) were assessed. The results revealed that most of these genes were upregulated upon LPS stimulation in a time-dependent manner, except for *Dlat* expression, which showed no significant difference (Figure 2A–C). Consistently, the levels of copper ions markedly increased after LPS treatment (Figure 2D).

To elucidate the effect of inhibiting cuproptosis on macrophage inflammation, TTM was administered to LPS-stimulated macrophages. Cell proliferation assays indicated that TTM inhibited cell death in macrophages stimulated with LPS (Figure 2E). Moreover, TTM treatment markedly suppressed the LPS-induced expression of copper transport-related genes and lipid acid pathway-related genes, while the expression of genes encoding the targets of lipoylated proteins showed no significant variation after TTM addition (Figure 2F–H and Figure S2). Furthermore, the protein expression of lipoylated proteins was upregulated in LPS-treated cells, and TTM partially reversed this effect (Figure 2J). To evaluate the role of copper or Elesclomol-induced cuproptosis in inflammation, TTM was applied to Cu or Elesclomol-stimulated macrophages. The data demonstrated that the levels of copper transport-related genes and lipid acid pathway-related genes were upregulated in Cu or Elesclomol-stimulated cells, which were reversed following TTM treatment (Figure 2I and Figure S2). These findings collectively suggest that LPS stimulation activates macrophage cuproptosis, which is subsequently inhibited by TTM treatment.

### 2.3. TTM Treatment Partially Alleviates LPS-Induced Cell Death by Enabling Macrophage Autophagy Flux

**Figure 3:**
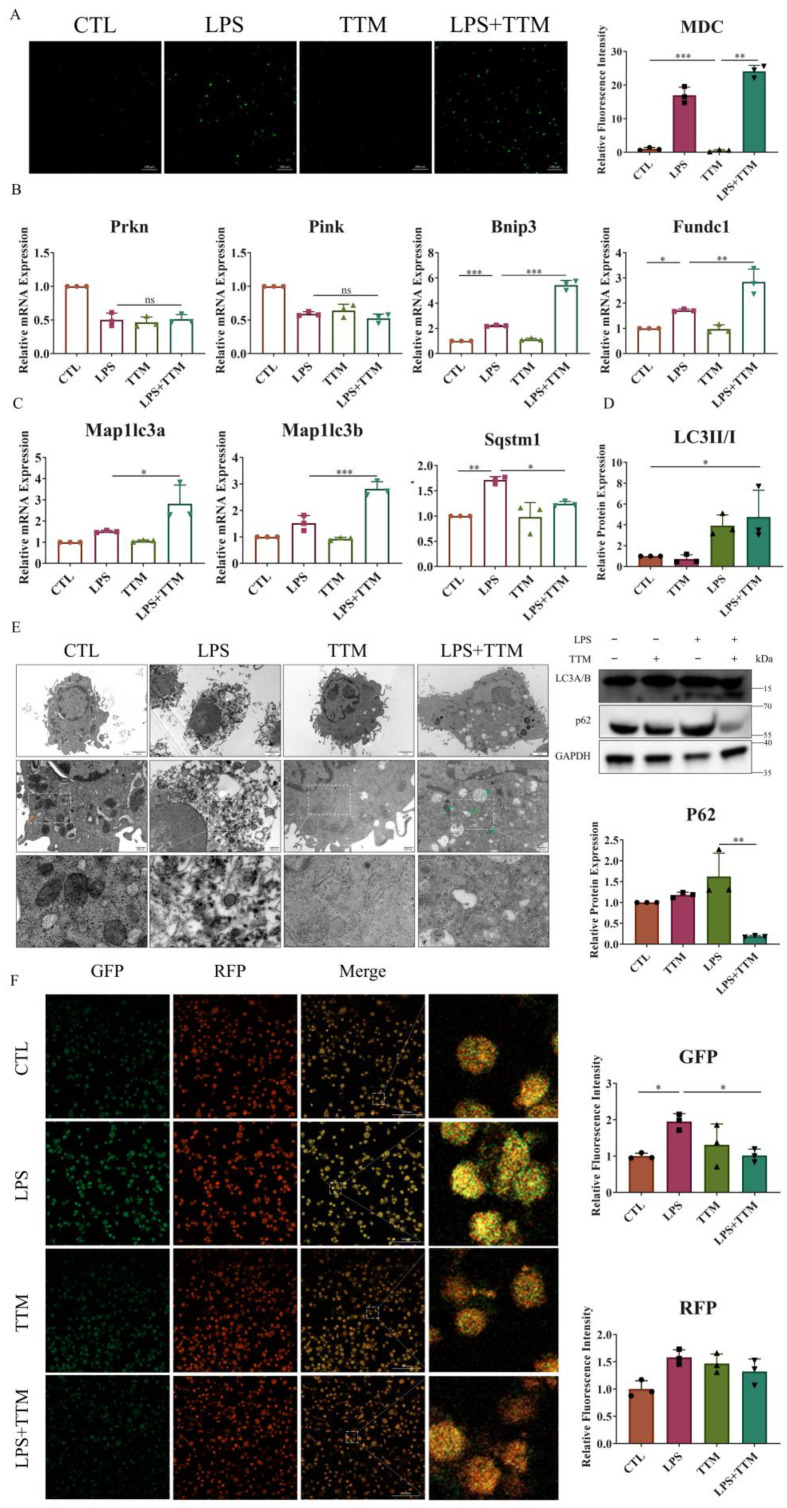
TTM treatment partially alleviates autophagy flux blockade in LPS-treated macrophages. (**A**) Images of the monodansylcadaverine staining were shown after the LPS (1 μg/mL) stimulation of BMDMs in the presence or absence of TTM (10 μM) at 72 h and quantified using imagej (20×, scale bar = 100 μm, *n* = 3); (**B**) The qRT–PCR analysis of mitophagy-related marker genes (*Prkn*, *Pink*, *Bnip3,* and *Fundc1*) were shown graphically at 72 h in the absence or presence of LPS (1 μg/mL) or TTM (10 μM) in BMDMs (*n* = 3); (**C**) The qRT–PCR analysis of autophagosome membrane formation-related marker genes (*Map1lc3a* and *Map1lc3b*) and the recruitment of specific cargo-related marker genes (*Sqstm1*) were shown graphically at 72 h in the absence or presence of LPS (1 μg/mL) or TTM (10 μM) in BMDMs (*n* = 3); (**D**) LC3A/B (as indicated by LC3II and LC3I) and P62 protein expression levels were measured via Western blotting using an anti-LC3A/B antibody and an anti-P62 antibody in BMDMs at 72 h and quantified using imagej (*n* = 3); (**E**) After treatment with LPS (1 μg/mL) and/or TTM (10 μM) in RAW264.7 cells for 72 h, the changes of cell ultrastructure were analyzed under a transmission electron microscope; The red arrows indicated mitochondria and the green arrows indicated autolysosomes (scale bar = 2 μm, 500 nm, *n* = 3); (**F**) RAW264.7 were pretreated with LPS (1 μg/mL) and TTM (10 μM) treatment for 72 h; Representative images of the abundance of LC3 puncta in the transient expression of GFP-RFP-LC3 RAW264.7 for groups and quantified using imagej (20×, scale bar = 100 μm, *n* = 3); All results are presented as the mean ± SD. * *p* < 0.05, ** *p* < 0.01, *** *p* < 0.001, and ns for no significant difference.

Cuproptosis induces protein toxic stress, leading to mitochondrial protein heteromerization, and potentially activating mitophagy to remove damaged mitochondria and prevent cell death [37]. To explore the effect of TTM on mitophagy, the number of autophagosomes was assessed in macrophages. The results revealed a significant increase in the number of autophagosomes following TTM treatment in LPS-stimulated macrophages (Figure 3A). Additionally, the mRNA expression levels of ubiquitin-independent mitophagy pathway-related markers (*Bnip3* and *Fundc1*), autophagy-related genes (*Map1lc3a* and *Map1lc3b*), and the protein expression of LC3A/B were markedly elevated in the LPS+TTM group when compared to the LPS group, while the expression of ubiquitin-dependent mitophagy pathway-related markers (*Prkn* and *Pink*) showed no significant variation (Figure 3B–D and Figure S3). These findings suggest the activation of ubiquitin-independent mitophagy in response to TTM treatment in LPS-induced macrophages.

Furthermore, the autophagy degradation gene *Sqstm1* was upregulated in LPS-treated macrophages, but was reversed following TTM treatment (Figure 3C). Similarly, the protein expression of P62 was increased in the LPS group and decreased in the LPS+TTM group, indicating the mitigating effect of TTM on LPS-induced autophagy flux inhibition (Figure 3D). The ultrastructural analysis of macrophages revealed that cells in the control group exhibited normal nuclear morphology, homogeneous nuclear chromatin, and well-defined mitochondrial structure, whereas macrophages in the LPS group showed nuclear shrinkage, chromatin condensation, and mitochondrial distortion (Figure 3E). After TTM treatment, macrophage ultrastructures were significantly normalized, with the presence of autolysosomes, compared to the LPS group (Figure 3E). To confirm the mitigating effect of TTM on autophagy flux inhibition, the GFP-RFP-LC3 experiment was utilized, showing that the reduced green spots in the LPS group increased again with the addition of TTM (Figure 3F). Overall, these results indicate that LPS-induced cuproptosis triggers autophagy flux blockade in macrophages, and the inhibition of cuproptosis using TTM alleviates this effect.

### 2.4. TTM Alleviates Autophagy Flux Blockade through Attenuating Lysosome Damage in LPS-Stimulated Macrophages

**Figure 4:**
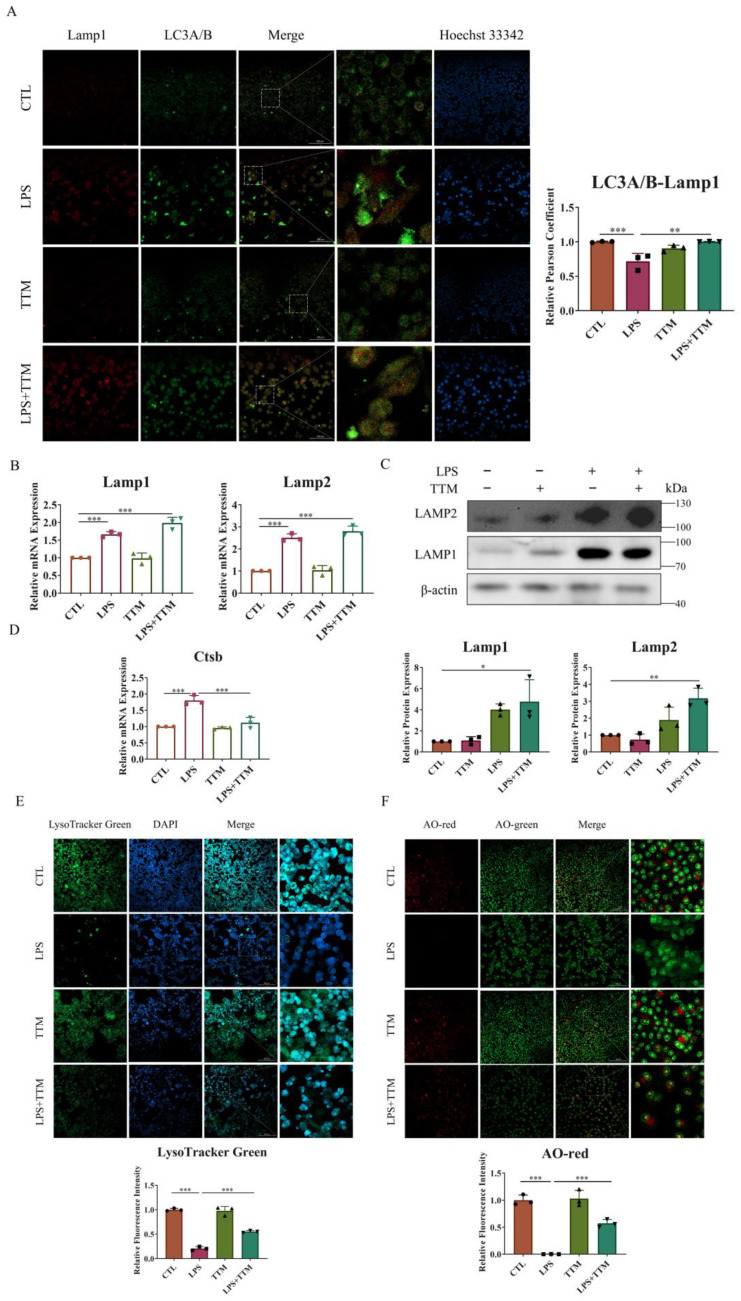
TTM alleviates the autophagy flux blockade induced by lysosome damage in LPS-stimulated macrophages. (**A**) Co-localization of LC3A/B and Lamp1 protein was observed using immunofluorescence staining in RAW264.7 cells for 72 h and quantified using imagej (20×, scale bar = 100 μm, *n* = 3); (**B**) The qRT-PCR analysis of *Lamp1* and *Lamp2* was shown graphically at 72 h in the absence or presence of LPS (1 μg/mL) or TTM (10 μM) in BMDMs (*n* = 3); (**C**) Lamp1 and Lamp2 proteins were measured using Western blotting using an anti-Lamp1 antibody and an anti-Lamp2 antibody in BMDMs for 72 h and quantified using imagej (*n* = 3); (**D**) The mRNA levels of *Ctsb* in BMDMs treated with LPS (1 μg/mL) or TTM (10 μM) were measured using qRT–PCR in BMDMs for 72 h (*n* = 3); (**E**) The RAW264.7 cells in the absence or presence of LPS (1 μg/mL) or TTM (10 μM) for 72 h were stained with Lyso-Tracker Green and quantified using imagej (20×, scale bar = 100 μm, *n* = 3); (**F**) The RAW264.7 cells in the absence or presence of LPS (1 μg/mL) or TTM (10 μM) for 72 h were stained with AO and quantified using imagej (20×, scale bar = 100 μm, *n* = 3); All results are presented as the mean ± SD. * *p* < 0.05, ** *p* < 0.01, *** *p* < 0.001.

Since autophagy is a lysosome-dependent self-degradation pathway, it is speculated that the cuproptosis-induced inhibition of autophagy flux may be due to lysosomal damage. To test this hypothesis, lysosomal function was assessed. Initially, our research focused on the fusion of lysosomes and autophagosomes through the immunofluorescence staining of LC3A/B and Lamp1 protein co-localization. As depicted in Figure 4A, a decreased level of fusion was observed in the LPS group, while a significant increase in merging was evident in the LPS+TTM group. Furthermore, the expression levels of lysosomal membrane proteins Lamp1 and Lamp2 were evaluated, with results showing the upregulation of both proteins, even in the presence of TTM, indicating that TTM treatment did not inhibit lysosome formation in LPS-stimulated macrophages (Figure 4B,C). Subsequently, the lysosomal pH was examined using Lyso-Tracker Green, with results showing weaker green fluorescence representing acidic pH in the LPS group compared to the control group, suggesting that LPS stimulation disrupted the acidic environment of lysosomes. Conversely, the green fluorescence in the LPS+TTM group was increased, indicating the partial restoration of lysosomal acidity following TTM treatment (Figure 4E). Finally, acridine orange (AO) dye was utilized to assess lysosomal permeability, with red fluorescence indicating oligomeric forms. The results demonstrated increased lysosomal permeability in LPS-stimulated cells, which was reversed following TTM (Figure 4F). In summary, these findings suggest that cuproptosis leads to the blockade of autophagy flux via impeding lysosomal–autophagosome fusion, disrupting lysosomal pH, and increasing lysosomal permeability in macrophages. To further investigate lysosomal damage induced by cuproptosis, the expression of cathepsin B, an important lysosomal hydrolase, was examined. The results indicated that the mRNA expression of *Ctsb* was upregulated upon LPS stimulation and reversed following TTM treatment (Figure 4D). Collectively, these results illustrate that lysosome damage is the underlying cause of the cuproptosis-induced blockade of autophagy flux in LPS-stimulated macrophages, and TTM can partially mitigate this effect.

### 2.5. TTM Treatment Alleviates Periodontitis by Inhibiting Cuproptosis in Mice

**Figure 5:**
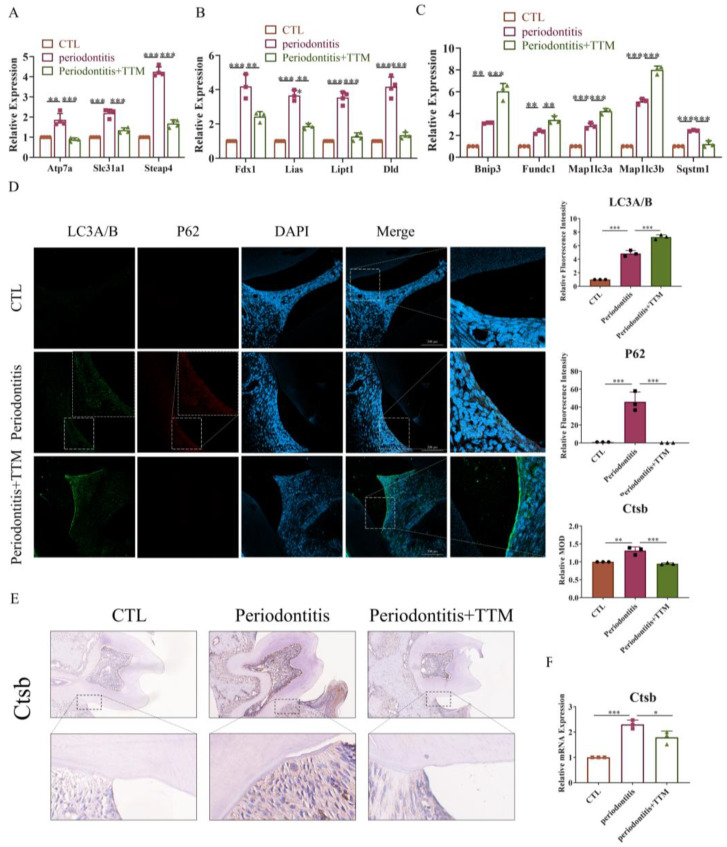
TTM treatment alleviates periodontitis by inhibiting cuproptosis in mice. (**A**) The mRNA expression of copper transport markers (*Atp7a*, *Slc31a1*, and *Steap4*) as measured using qRT-PCR in periodontal tissues (*n* = 3); (**B**) The mRNA expression of cuproptosis markers (*Fdx1*, *Lias*, *Dld*, and *Lipt1*) as measured using qRT-PCR in periodontal tissues (*n* = 3); (**C**) The mRNA expression of mitophagy markers (*Bnip3*, *Fundc1*, *Map1lc3a*, *Map1lc3b*, and *Sqstm1*) as measured using qRT-PCR in periodontal tissues (*n* = 3); (**D**) Representative immunofluorescence images of LC3A/B and P62 in the periodontal tissue of the CTL, periodontitis, and periodontitis + Tetrathiomolybdate (periodontitis +TTM) groups (20×, scale bar = 200 μm, *n* = 3); (**E**) Immunohistochemistry staining of cathepsin B in the periodontal tissues (20×, scale bar = 200 μm, *n* = 3); (**F**) The mRNA expression of Ctsb as measured using qRT-PCR in periodontal tissues (*n* = 3); All results are presented as the mean ± SD. * *p* < 0.05, ** *p* < 0.01, *** *p* < 0.001.

To confirm the impact of macrophage cuproptosis on periodontitis in vivo, TTM was administered to periodontitis mice via gavage, and the bilateral maxillae were dissected to obtain gingival and periodontal membranes for study. As depicted in Figure 5A,B, the elevated levels of copper transport-related genes and lipid acid pathway-related genes observed in the periodontitis group were attenuated following TTM treatment. The results pertaining to mitophagy-related markers indicated that the expression levels of mitophagy pathway-related genes (*Bnip3* and *Fundc1*) and autophagy-related genes (*Map1lc3a* and *Map1lc3b*) were significantly upregulated in the periodontitis+TTM group when compared with the periodontitis group (Figure 5C). Moreover, the findings from LC3A/B-P62 colocalization analyses further confirmed that TTM could alleviate the blockade of autophagy flux in periodontitis mice (Figure 5D). Additionally, the results of immunohistochemical staining suggested that the levels of cathepsin B protein significantly increased in the periodontitis group, which were partially reversed following TTM treatment (Figure 5E). Consistently, the mRNA expression of *Ctsb* significantly increased in the periodontitis group, while exhibited a decrease in the periodontitis+TTM group (Figure 5F). All the aforementioned results were consistent with those observed in vitro, indicating that TTM alleviated autophagy flux blockade and lysosome damage by inhibiting cuproptosis, thereby attenuating periodontitis.

## 3. Discussion

Periodontitis is a common infectious disease that progressively impairs the supportive structures of the teeth, involving complex dynamic interactions among bacterial pathogens and host immune responses. In rodents with ligatures placed around the teeth, bacteria initiates inflammation and periodontal bone loss, similar to the development process of human periodontal disease [1,2,3]. The rodent model was thus widely used to investigate host–bacteria interactions related to periodontal diseases [38]. In the present study, a ligature-induced periodontitis model was established to investigate the role of cuproptosis-related macrophage inflammation in periodontal disease.

Research indicates increased copper concentrations in the saliva of individuals with periodontitis, which stabilize after treatment [39,40,41]. When inflammation occurs, there is an increase in serum copper levels, leading to oxidative stress and inflammation [34]. The pro-inflammatory effect of copper is attributed to its role in stimulating ROS generation through the Fenton and Haber–Weiss reactions [42,43]. Fascinatingly, issues like copper imbalance, oxidative stress, and inflammation frequently occur in numerous chronic illnesses [34]. Aligning with these results, our research identified elevated copper concentrations in the periodontal tissues of individuals with periodontitis. Yet, it is still uncertain if an abundance of copper triggers cell cuproptosis in cases of periodontitis. Examining single-cell sequencing data from individuals with periodontitis showed gene expression linked to cuproptosis in their periodontal tissues. Copper chelators are generally used to regulate the cellular copper homeostasis and suppress cell cuproptosis [24,27,28]. Following this, a mouse model of periodontitis, when treated with copper chelator TTM, exhibited decreased bone degradation and a reversal in the increase in proteins associated with cuproptosis, indicating the role of inhibiting cuproptosis in the advancement of periodontitis. The simultaneous presence of lipoic acid, a cuproptosis indicator, and F4/80, a macrophage marker, suggests the involvement of macrophages in triggering cuproptosis during periodontitis, prompting the further exploration of inhibiting cuproptosis through TTM mechanisms in vitro.

The recruitment of macrophages is directly linked to the intensity of periodontal diseases [44]. Infiltrated macrophages experience a range of regulatory cell deaths, leading to the release of inflammatory substances that exacerbate periodontal disease [23,45]. Through the release of IL-1β, macrophage pyroptosis hastens the aging of periodontal tissue [22]. Through the *Ampk/Sirt1/Nf-κb* pathway, glycolytic reprogramming triggers macrophage pyroptosis in periodontitis lesions, intensifying inflammation and bone degradation [15]. Our study of cuproptosis’s impact on macrophage inflammation involved analyzing RAW264.7 cells and BMDMs activated by LPS. Post LPS treatment, there was a rise in intracellular copper ions and lipoylation-related proteins, accompanied by an increase in genes associated with cuproptosis. Treatment with TTM resulted in the suppression of cell death, curtailed gene expression linked to cuproptosis, and reduced levels of lipoylated proteins. Additionally, TTM counteracted the increased activity of genes linked to cuproptosis in copper-stimulated macrophages, indicating their role in alleviating cuproptosis associated with periodontitis. Nonetheless, further studies are required to clarify the mechanisms and effects of TTM in periodontitis, particularly its influence on mitochondrial quality control mechanisms such as mitophagy.

Autophagy can be triggered by copper via multiple routes [46,47,48]. Yet, it is still uncertain if cuproptosis triggers autophagy. The phenomenon of cuproptosis arises when an abundance of copper attaches to lipoylated proteins, causing the misfolding of mitochondrial proteins and possibly triggering mitophagy to preserve mitochondrial balance [24,49]. In this study, treatment with TTM enhanced the activation of mitophagy in macrophages stimulated by LPS, indicating that cuproptosis triggered by LPS stimulates mitophagy. TTM enhanced the creation of autophagosomes and the initiation of mitophagy in LPS-treated macrophages, while the expression of the autophagy receptor P62/*Sqstm1* rose after LPS activation, but reverted post TTM treatment, suggesting that cuproptosis impedes autophagy flux, while TTM partially restores this effect. Additionally, the GFP-RFP-LC3 imagery revealed a decrease in autophagy flux following LPS stimulation, which was subsequently reinstated through TTM therapy, suggesting that cuproptosis triggered by LPS hinders the autophagy flux in macrophages, a process which TTM mitigates.

The process of mitophagy, reliant on lysosomes for autodegradation, necessitates the maintenance of lysosomal functionality [50,51,52]. The obstruction of autophagy flux in macrophages treated with LPS, triggered by cuproptosis, stemmed from lysosome damage, evidenced by the hindered fusion between autophagosomes and lysosomes, impaired acidity in lysosomes, and heightened permeability of lysosomes, all of which were counteracted by TTM treatment in our research. Furthermore, TTM inhibited the release of cathepsin B in LPS-stimulated macrophages. Consistent with the above findings in vitro, the results of the in vivo experiments demonstrated that TTM could modulate the cuproptosis/mitophagy/lysosome axis in periodontitis. Upcoming studies aim to investigate the lysosomal damage processes linked to macrophage cuproptosis in periodontitis, anticipated to be detailed in future publications.

## 4. Materials and Methods

### 4.1. Acquisition of Human Periodontal Tissue Samples and Mouse Models of Periodontitis

Subjects were randomly selected from the oral and maxillofacial surgery and periodontal departments for the study (*n* = 6, 3 healthy patients, 3 patients with periodontitis). All subjects had no systemic diseases such as blood diseases, diabetes, and rheumatoid arthritis, had not taken hormone drugs or antibiotics in the last 3 months, and had undergone no periodontal surgery within the last 6 months. Female subjects were not pregnant or lactating. The inclusion criteria of chronic periodontitis were mainly based on the classification of periodontal diseases formulated by the American Academy of Periodontology. The study protocol was reviewed and approved by the Medical Ethics Committee of the Dental Hospital (KOEC-2022-121-01), ensuring that the rights of the participants are protected. The study was carried out in accordance with the 1964 Declaration of Helsinki, which was revised in 2013. When eligible subjects underwent relevant surgery, the full thickness gingival tissue, including the epithelial tissue and subcutaneous connective tissue, with a length of about 3 mm and a width of about 2 mm, was cut from the gingival margin to the root. The gingival tissue specimens were placed in phosphate buffer saline (PBS) or an alpha-minimum essential medium (α-MEM medium).

The animal study followed the ARRIVE (Animal Research: Reporting of In Vivo Ex-periments) 2.0 guidelines. Male C57BL/6 mice, aged between five and eight weeks and weighing 20–25 g, were obtained from the Animal Center at Sun Yat-sen University. All experimental procedures were approved by the Animal Care and Use Committee of Sun Yat-sen University (Approval No.: SYSU-IACUC-2023-000134).

### 4.2. Data Acquisition and Cell Clustering

ScRNA-seq information for individuals with periodontitis (GSE164241) was sourced from the GEO database. Initially, the R package (R 4.3.2)’s Seurat was employed for quality assurance. Subsequently, the residual cells were utilized in the following research. Utilizing the FindVariableGenes feature in the Seurat package (5.0.1), the leading 2000 variable genes were chosen. Following this, the execution of principal component analysis (PCA) and t-SNE took place. A variety of cell types were pinpointed through the analysis of markers. The analysis of cell type abundance uncovered the percentage of cell clusters in the periodontitis group, with feature plots illustrating the expression of genes related to cuproptosis in this group.

### 4.3. Cell Culture

RAW 264.7 macrophages were acquired from the American Type Culture Collection (ATCC, Manassas, VA, USA). The cells underwent cultivation inα-MEM medium (Gibco, New York, NY, USA), enriched with 10% fetal bovine serum (FBS; Gibco, Carlsbad, CA, USA), maintained at 37 °C in a moisture-rich environment of 5% CO_2_ and 95% air. Upon attaining 80% confluence, the cells were collected with cell scrapers and then subcultured at a ratio of 1:3.

Bone marrow cells were extracted from male mice, C57BL/6 (Jax 000664), on the 28th day after birth (Animal Center of Sun Yat-sen University). Mice were sacrificed under anesthesia, and soaked in 75% ethanol for 30 min to be sterilized and disinfected. Both femurs and tibia were taken and cut, and the bone marrow was blown out of the medium with a syringe. Then, the medium was repeatedly blown, transferred to a 15 mL centrifuge tube, centrifuged at 1500 rpm for 5 min, and the supernatant was discarded. Cells extracted from each mouse underwent a four-day cultivation process to produce bone marrow-derived macrophages (BMDMs) in the α-MEM medium, enriched with 10% fetal bovine serum and 30 ng/mL macrophage-stimulating factor (M-CSF; Sino Biological, Beijing, China).

### 4.4. Cell Stimulation

The cells underwent incubation in either a 6-well or 12-well culture dish for 72 h. After adhering to the plate, they received an induction of 1 μg/mL LPS (InvivoGen, San Diego, CA, USA) at intervals of 0, 12, 24, 48, and 72 h. Additionally, the control group received the α-MEM medium.

In the salvage experiment, cells underwent treatment with TTM (Beyotime, Shanghai, China; 10 μM), Cupric chloride (CuCl_2_) (Beyotime, Shanghai, China; 50 μM), Elesclomol (Macklin, Woking, UK, 200 nM) or CA-074 Me (Beyotime, Shanghai, China; 1 μM), followed by a 72 h stimulation with 1 μg/mL LPS. Cells either not activated with LPS or exposed to inhibitors served as the control.

### 4.5. Cell Counting Kit-8 (CCK8) Assay

Cell proliferation was assessed using the CCK8 assay (Dojindo, Kumamoto, Japan) according to the guidelines provided by the manufacturer. After incubating the cells for 0, 12, 24, 48, or 72 h, the clear liquid was supplemented with the α-MEM medium containing CCK-8 and incubated for another 2 h. To measure optical density (OD) at 450 nm, an automatic microplate reader (Sunrise, Tecan, Hombrechtikon, Switzerland) was used.

### 4.6. Quantitative Reverse Transcription PCR (RT-qPCR)

RNA extraction from cells was performed with RNAzol (MRC, OH, USA), followed by purification as per the manufacturer’s guidelines. Utilizing a PrimeScriptTM RT reagent kit (Takara, Kyoto, Japan), a microgram of RNA underwent reverse transcription into cDNA. The qRT-PCR process utilized a LightCycler 480 setup equipped with SYBR Green I Master Mix (Roche, Basel, Switzerland). *β-actin*, a housekeeping gene, served as the benchmark gene. The comparative expression levels of the targeted genes were standardized against the geometric mean of *β-actin* expression. The sequences of primers can be found in Table S1.

### 4.7. Western Blotting Analysis

Protein was isolated utilizing Radio Immunoprecipitation Assay Lysis buffer (RIPA; Beyotime, Haimen, China) and quantified with a BCA kit (ComWin Biotech, Beijing, China). Twenty-five micrograms of protein underwent 10% SDS-polyacrylamide gel electrophoresis and were then moved onto polyvinylidene fluoride membranes (PFM; Millipore, Billerica, MA, USA). Membranes underwent blocking using 5% bovine serum albumin for an hour, followed by incubation with primary antibodies LC3A/B (Wanleibio, Shenyang, China, WL01506, 1:1000), P62 (Wanleibio, Shenyang, China, WL02385, 1:1000), Lamp1 (Wanleibio, Shenyang, China, WL02419, 1:1000), Lamp2 (Wanleibio, Shenyang, China, WL02761, 1:1000), β-actin (Cell Signaling Technologies, Danvers, MA, USA, 4967, 1:1000), and Gapdh (Cell Signaling Technologies, Danvers, MA, USA, 2118, 1:1000). Post-washing, the membranes were localized with secondary antibodies (Cell Signaling Technologies, Danvers, MA, USA, 7074, 1:2000) for 1 h. The luminescent liquid (liquid A and liquid B) was configured at a rate of 1:1, and the appropriate amount of luminescent liquid was absorbed with a pipette to cover the Polyvinylidene fluoride (PVDF) membrane, and the visualization of the proteins was achieved through an advanced chemiluminescence system (Millipore, Billerica, MA, USA).

### 4.8. Cell Immunofluorescence (IF) Staining

For identifying the simultaneous presence of Lipoic acid-F4/80 and LC3-Lamp1, cells were cultured in 12-well Petri dishes with cell slides and fixed with a 4% Paraformaldehyde Fix Solution (Beyotime, Shanghai, China) for 8 min, followed by permeabilization using 0.1% Triton X-100 in PBS for 15 min, blocking with 2% bovine serum albumin (BSA) in PBS for an hour. Subsequently, the cells were treated with either a LC3A/B antibody (Wanleibio, Shenyang, China, WL01506, 1:250) or a Lipoic acid antibody (Abcam, Cambridge, UK, ab58724, 1:250) at 4 °C throughout the night, and then with an Alexa Fluor^®^ 488-conjugated secondary antibody (Cell Signaling Technologies, Danvers, MA, USA, 8878, 1:500) for an hour. Following a triple rinse with PBS, the cells were treated with either a F4/80 antibody (Cell Signaling Technologies, Danvers, MA, USA, 70076, 1:250) or a Lamp1 antibody (Wanleibio, Shenyang, China, WL02419, 1:250) in PBS for 12 h, and finally with an Alexa Fluor^®^ 647-conjugated secondary antibody (Cell Signaling Technologies, Danvers, MA, USA, 8940, 1:500) for 1 h. The nuclei were then stained with 4’,6-diamidino-2-phenylindole (DAPI) or bisBenzimide H 33342 (Hochest33342). Illustrative images were captured using a confocal microscope (LSM 980, Zeiss, Oberkochen, Germany) equipped with a ×20 objective lens. Immunostained cells were observed using an excitation wavelength of 488 nm to visualize Lipoic acid and LC3A/B,647 nm to visualize F4/80 and Lamp1, and 405 nm to visualize the nucleus. The average fluorescence intensity was measured using ImageJ v1.54h software (National Institute of Health, Bethesda, MD, USA).

### 4.9. Monodansylcadaverine (MDC) Staining

Following the specified treatment protocols, the cells were transferred to a 6-well plate for overnight cultivation, then stained using 0.05 mM MDC (Leagene, Beijing, China) at 37 °C for half an hour. After being rinsed thrice with PBS, the cells underwent fixation using 4% paraformaldehyde at an ambient temperature for 10 min and were promptly examined using a fluorescence microscope (LSM 980, Zeiss, Oberkochen, Germany). MDC-immunology-positive cells were observed using an excitation wavelength of 488 nm. The average fluorescence intensity was measured using ImageJ v1.54h software (National Institute of Health, Bethesda, MD, USA).

### 4.10. Monitoring and Measuring Autophagy Flux

Cellular transfection was performed using the RFP-GFP-LC3 lentivirus (Lifeqho, Shanghai, China). Cells underwent transfection for a minimum of 24 h before being detected. The cells underwent inoculation in glass-bottomed culture dishes, followed by a 48 h infection with the RFP-GFP-LC3 lentivirus, stabilization in 4% paraformaldehyde, and subsequent DAPI staining for nuclei examination. Following the specified treatment parameters, confocal laser scanning microscopy (LSM 980, Zeiss, Oberkochen, Germany) was employed to track the autophagic flux. An excitation wavelength of 488 nm was used to visualize the GFP, while the RFP was visualized using an excitation wavelength of 647 nm. ImageJ v1.54h software (National Institute of Health, Bethesda, MD, USA) was employed to measure the average fluorescence intensity.

### 4.11. Acridine Orange Staining (AO) and Lyso-Tracker Green Staining (LTG)

The cells underwent incubation with either AO ((Macklin, Woking, UK, 5 μg/mL) or LTG (ThermoFisher, Massachusetts, USA, 100 nM) working solutions at a temperature of 37 °C for a duration of 30 min. The slides underwent a cleansing process using PBS and were examined using a confocal microscope (LSM 980, Zeiss, Oberkochen, Germany) equipped with a × 20 lens. We use an excitation wavelength of 488 nm to visualize the LTG and 405 nm to visualize the nucleus. When observing AO-immunology-positive cells, the excitation wavelength was 488 nm, while the data were obtained at two separate emission wavelengths (505–560 nm, 590–690 nm). The average fluorescence intensity was measured using ImageJ v1.54h software (National Institute of Health, Bethesda, MD, USA).

### 4.12. Transmission Electron Microscopy (TEM)

Following a 72 h regimen as per the experimental plan, cells underwent treatment with 2.5% glutaraldehyde in a sodium vanadate buffer (0.05 M) for over 2 h, subsequently stabilized using 1% phosphorus tetroxide and colored with uranyl acetate and lead citrate. Samples of the sections were processed and visualized using a TEM device (H7500, Hitachi, Tokyo, Japan).

### 4.13. Measurement of Intracellular Copper

Cells were cultured in 6 cm plates throughout the night and exposed to LPS for 72 h. Subsequently, these cells were gathered, reconstituted with 120 μL ddH_2_O, and ultrasonically fragmented to gather intracellular copper for identification, following the guidelines provided by the manufacturer (Elabscience, Wuhan, China).

### 4.14. Ligature-Induced Periodontitis Mice

This animal study followed the ARRIVE (Animal Research: Reporting of In Vivo Experiments) 2.0 guidelines. Male C57BL/6 mice, aged between five and eight weeks and weighing 20–25 g, were acquired from Sun Yat-sen University’s Animal Center. The mice were nurtured in an environment regulated by temperature and humidity, following a 12 h cycle of light and darkness. Every experimental procedure has already been ethically approved by Sun Yat-sen University’s Animal Care and Use Committee (SYSU-IACUC-2023-000134). The mice were arbitrarily segmented into three categories (*n* = 5) as follows: a control group, a group with periodontitis, and a group combining periodontitis and TTM. The animal model of periodontitis mice was established by placing a 5-0 ligature around the cervix of the first and second maxillary molars. The stomaching of the control reagent or TTM was performed bi-daily, starting from the day of model establishment. Following a 10 day period of ligature application, the mice were euthanized to collect and stabilize their maxilla. TTM, produced by Beyotime in Shanghai, China, served as a copper-chelating agent, dissolved in a mixture containing 40% PEG300, 5% Tween-80, 5% DMSO, and 50% saline. As a control measure, the reagent lacking TTM was employed. The mouse model for periodontitis caused by ligature was developed based on earlier research [53]. Post μCT scanning, the tissues underwent decalcification before their preparation for histological examination.

### 4.15. Microcomputed Tomography (Micro-CT) Analysis

Mouse maxillaries were extracted and meticulously separated from all soft tissues for conducting 3D micro-CT scans (Scano Medical AG, Bassersdorf, Switzerland). The scanning of the samples was conducted, followed by the reconstruction of 3D image sequences. For assessing the extent of bone degradation, the straight-line span from the ABC to CEJ was measured at six different palatal locations on upper molar teeth in three-dimensional μCT maxillae images. The ABC is the highest line of the alveolar bone, and the CEJ is the anatomical region where the cementum meets the enamel at the cervical region of the tooth. Both the ABC and CEJ were observed using ImageJ v1.54h software (National Institutes of Health, Bethesda, MD, USA). The linear distance from the ABC to the CEJ was determined at the upper first and second molar teeth in 3D micro-CT images of maxillae, showing the absorption of the alveolar bone.

### 4.16. Quantification of Cu Via ICP-MS

Initially, the sample was subjected to a preliminary pre-digestion phase, involving a treatment using a blend of 65% HNO_3_ and 30% H_2_O_2_. Subsequently, the specimen underwent boiling in microwave acid digestion machinery at 180 °C for 20 min and was diluted with deionized water. Then, the specimen underwent a two-hour heating process in exposed containers on a hot plate, being kept at a steady temperature of 150 °C. Post digestion, the supernatant underwent filtration using a 0.5 μm filter to isolate any residual undissolved particles. Ultimately, the mixture was cooled to an ambient temperature and then diluted to a total volume of 3 mL using 2% HNO_3_ for determination via ICP-MS. The accumulation of Cu in the samples was gauged using an ICP-MS (Agilent, ICPMS 7700 system, Santa Clara, CA, USA).

### 4.17. Tissue Histologic, Immunohistochemical Staining, and Immunofluorescence Staining

The maxillaries were preserved in 4% paraformaldehyde at a temperature of 4 °C for a duration of 36 h, encased in paraffin, and sliced into slides of 4 μm thickness. The sections underwent staining using hematoxylin–eosin (HE), adhering to the histological post-deparaffinization and rehydration assessment protocol. The relative colocalization Pearson coefficient was measured using ImageJ v1.54h software (National Institute of Health, Bethesda, MD, USA).

To achieve tissue immunohistochemical and immunofluorescence staining, the sections underwent incubation with a pepsin solution to retrieve antigens. Following treatment with 3% hydrogen peroxide, the slides underwent blocking using 5% BSA and were then left to incubate with the primary antibody at 4 °C overnight. For tissue immunohistochemical staining, the slides underwent a 1 h incubation with an HRP-linked secondary antibody, followed by a counterstain of the nuclei using hematoxylin. A digital scanner (Leica Biosystems, Buffalo Grove, IL, USA) was used to capture the sections. For tissue immunofluorescence staining, the sections were incubated with a pepsin solution. The slides were incubated with an Alexa Fluor^®^ 488-conjugated secondary antibody (1:500 dilution) for 1 h. After washing three times with PBS, the slides were incubated with another primary antibody at 4 °C for 12 h, then treated with the Alexa Fluor^®^ 647-conjugated secondary antibody (1:500 dilution) for 1 h, and the nuclei were counterstained with DAPI. Recordings of the sections were made using a digital scanner (Leica Biosystems, Buffalo Grove, IL, USA) and a confocal microscope (LSM 980, Zeiss, Oberkochen, Germany). The immunology-positive cells were observed using an excitation wavelength of 488 nm to visualize Lipoic acid and LC3A/B, 647 nm to visualize F4/80 and P62, and 405 nm to visualize the nucleus. The average fluorescence intensity was measured using ImageJ v1.54h software (National Institute of Health, Bethesda, MD, USA).

### 4.18. Statistical Analysis

Each experiment was conducted a minimum of three times, with the results displayed as the mean ± standard deviation (SD). All data were analyzed using GraphPad Prism 8. The Student’s *t*-test facilitated the comparison between two groups following a normal distribution, while a one-way variance analysis was utilized for the experimental group comparisons. A *p*-value below 0.05 was deemed statistically significant.

## 5. Conclusions

To sum up, the research revealed the induction of cuproptosis in inflammatory macrophages and the inhibition effect of TTM on cuproptosis via both in vivo and in vitro studies. Mechanistically, TTM treatment triggered mitophagy and alleviated autophagy flux blockade and lysosome damage via inhibiting macrophage cuproptosis, thereby attenuating periodontitis. Future research will delve deeper into the effects of copper-chelating agents and ionophores such as BCS and Elesclomol on the periodontitis, formulating novel treatment approaches to adjust cuproptosis for periodontitis. The results suggest that macrophage cuproptosis is vital in the inflammatory development of periodontitis, suggesting an innovative method to tackle macrophage dysfunction caused by inflammation through TTM.

## Data Availability

The data used in this study are all available from the GEO database (https://www.ncbi.nlm.nih.gov/geo/ (accessed on 10 March 2024)), Expression profiling by high throughput sequencing (number: GSE164241) at https://www.ncbi.nlm.nih.gov/geo/query/acc.cgi?acc=GSE164241 (accessed on 10 March 2024).

## Supplementary Materials

The supporting information can be downloaded at: https://www.mdpi.com/article/10.3390/ijms25115890/s1.
