# Supplementary material for: Copper Chelation Therapy Attenuates Periodontitis Inflammation through the Cuproptosis/Autophagy/Lysosome Axis
Source: Int J Mol Sci. 2024 May 28;25(11):5890. doi: 10.3390/ijms25115890 (PMC11172687; doi:10.3390/ijms25115890)
Supplement: Supplementary file 1 [file ijms-25-05890-s001.zip › ijms-2971089-supplementary/Supplementary Material.pdf]

Supplemental Materials & Methods

A

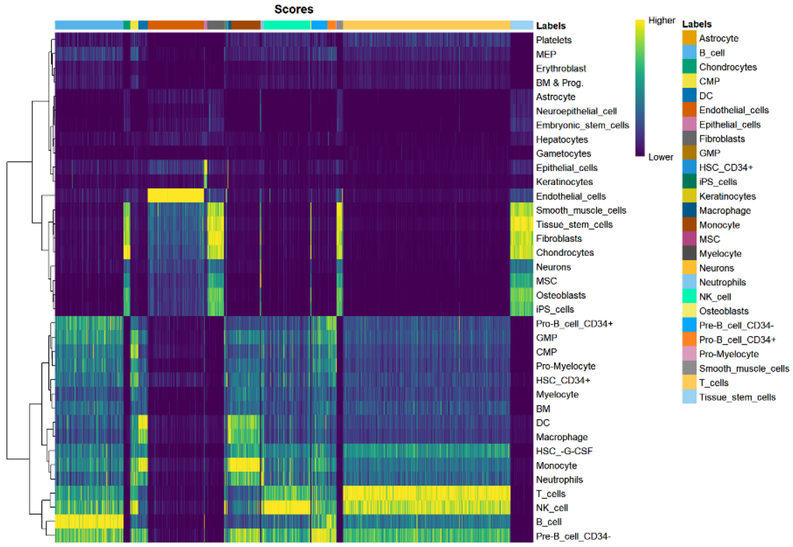
Scores
Labels
Higher
Lower
Platelets
MEP
Erythroblast
BM & Prog.
Astrocyte
Neuroepithelial_cell
Embryonic_stem_cells
Hepatocytes
Gametocytes
Epithelial_cells
Keratinocytes
Endothelial_cells
Smooth_muscle_cells
Tissue_stem_cells
Fibroblasts
Chondrocytes
Neurons
MSC
Osteoblasts
iPS_cells
Pro-B_cell_CD34+
GMP
CMP
Pro-Myelocyte
HSC_CD34+
Myelocyte
BM
DC
Macrophage
HSC_-G-CSF
Monocyte
Neutrophils
T_cells
NK_cell
B_cell
Pre-B_cell_CD34-
Astrocyte
B_cell
Chondrocytes
CMP
DC
Endothelial_cells
Epithelial_cells
Fibroblasts
GMP
HSC_CD34+
iPS_cells
Keratinocytes
Macrophage
Monocyte
MSC
Myelocyte
Neurons
Neutrophils
NK_cell
Osteoblasts
Pre-B_cell_CD34-
Pro-B_cell_CD34+
Pro-Myelocyte
Smooth_muscle_cells
T_cells
Tissue_stem_cells

B

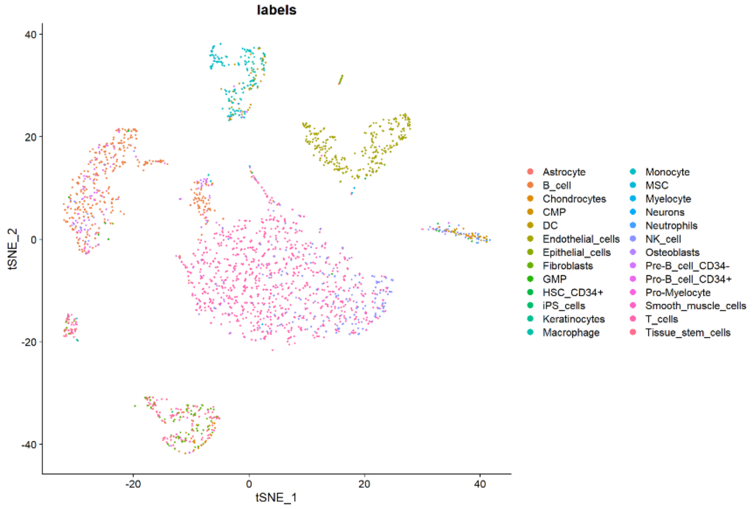
labels
40
20
0
-20
-40
tSNE_2
-20
0
20
40
tSNE_1
Astrocyte
B_cell
Chondrocytes
CMP
DC
Endothelial_cells
Epithelial_cells
Fibroblasts
GMP
HSC_CD34+
iPS_cells
Keratinocytes
Macrophage
Monocyte
MSC
Myelocyte
Neurons
Neutrophils
NK_cell
Osteoblasts
Pre-B_cell_CD34-
Pro-B_cell_CD34+
Pro-Myelocyte
Smooth_muscle_cells
T_cells
Tissue_stem_cells

C

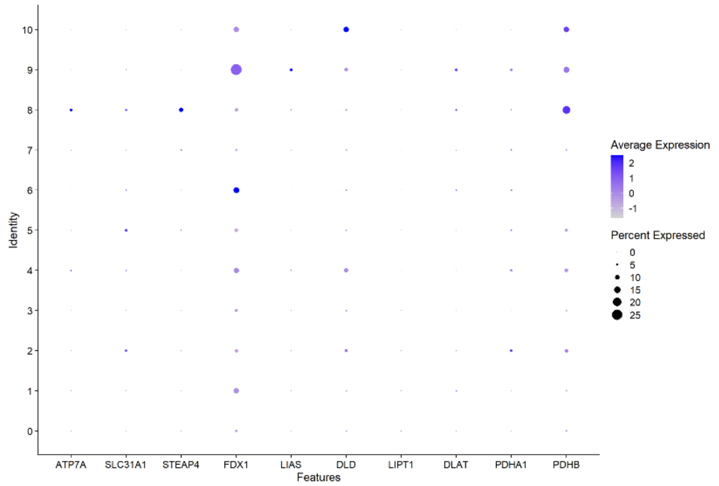
10
9
8
7
6
5
4
3
2
1
0
Identity
ATP7A
SLC31A1
STEAP4
FDX1
LIAS
DLD
LIPT1
DLAT
PDHA1
PDHB
Features
Average Expression
2
1
0
-1
Percent Expressed
0
5
10
15
20
25

D

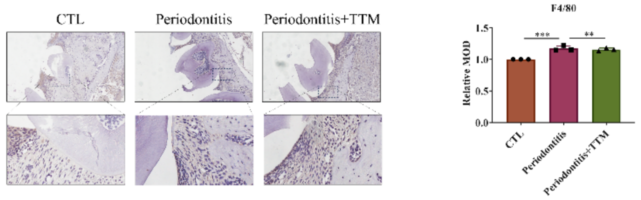
CTL
Periodontitis
Periodontitis+TTM
F4/80
Relative MOD
1.5
1.0
0.5
0.0
***
**
CTL
Periodontitis
Periodontitis+TTM

Supplemental Fig. 1. Single cell data analysis of cuproptosis-related gene expression in periodontal tissues of patients with periodontitis. (A) Heat map of the single-cell profile colored by 'single' R package. (B) TSNE plot of the single-cell profile colored by 'single' R package. (C) The bubble map shows the expression of cuproptosis-related genes; (D) Characteristic images of immunohistochemical staining of F4/80 in the periodontal tissue slices (20×, scale bar = 200 μm, n = 3). ** $p < 0.01$, *** $p < 0.001$

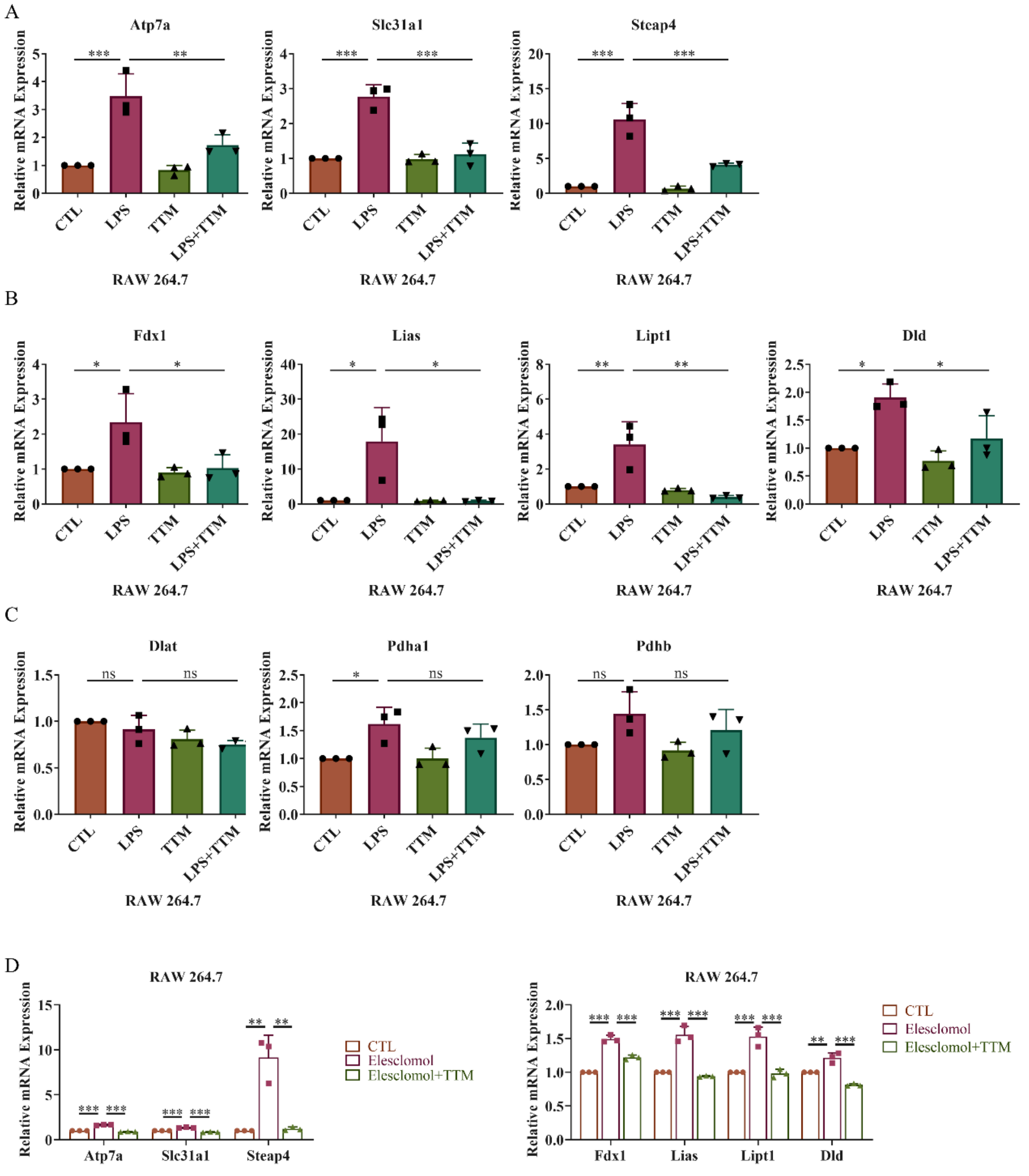

Supplemental Fig. 2. The effect of TTM on LPS-induced cuproptosis in RAW264.7 macrophages. (A) The mRNA expression of copper transport-related genes (ATP7A, SLC31A1, and STEAP4) was quantified by qRT–PCR in RAW264.7 cells (n = 3). (B) The mRNA expression of lipid acid pathway-related genes (FDX1,

LIPT1, LIAS and DLD) was quantified by qRT–PCR in RAW264.7 cells (n = 3). (C) The mRNA expression of lipoacylated protein targets genes (DLAT, PDHA1 and PDHB) was quantified by qRT–PCR in RAW264.7 cells (n = 3). (D) qRT-PCR analysis of the mRNA expression of copper transport-related genes (Atp7a, Slc31a1, and Steap4) and lipid acid pathway-related genes (Lipt1, Lias and Dld) in RAW264.7 cells treated with Elesclomol (200 nM) in the presence or absence of TTM (10 μM) for 72 h (n = 3). * $p < 0.05$, ** $p < 0.01$, *** $p < 0.001$, and ns for no significant difference.

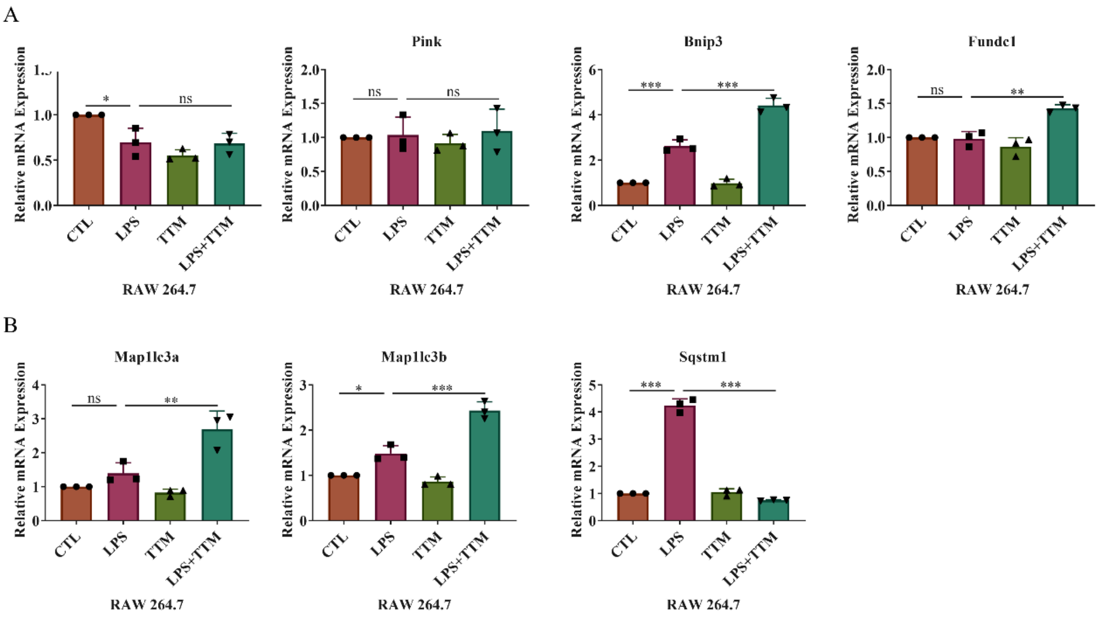

Supplemental Fig. 3. Images of the stained autophagic vesicles and the qPCR analysis of mitophagy-related marker genes in RAW264.7 macrophages. (A) The quantitative real-time PCR analysis of mitophagy-related marker genes (PRKN, PINK, BNIP3 and FUNDC1) was shown graphically at 72 hours in the absence or presence of LPS or TTM in RAW264.7 cells (n = 3). (B) The quantitative real-time PCR analysis of autophagosome membrane formation-related marker genes (MAPILC3A and MAPILC3B), and the recruitment of specific cargo-related marker genes (SQSTM1) was shown graphically at 72 hours in the absence or presence of LPS or TTM in RAW264.7 cells (n = 3). * $p < 0.05$, ** $p < 0.01$, *** $p < 0.001$, and ns for no significant difference.

Supplemental Table 1. Primer sequence

Primer Name	5′–3′ Forward	5′–3′ Reverse

β-actin	CATACCCAAGAAGGAAGGCTGG	GCTATGTTGCTCTAGACTTCGAGC
Fdx1	TCAAGAACCGAGATGGCGAG	GTTCCCTCACACGCACCAAA
Lias	TCCCTCGATCCCTTGACACT	CCATGTACTCTTGTCCGGG
Steap4	GGCTCTCCAGTCAGGAACACT	GGTGAGCCCAAGAGTACGAG
Atp7a	GGAAACCTACTTTCCCGGCTA	TTGGAGTGGCTAGTCCCAGT
Slc31a1	GGGCTTACCCTGTGAAGACTTT	GTGGTGAGGTGGCATGGTAA
Lipt1	AACTGCGTCTAGAGGGCTGA	AAGTGGAATTAGCATGCTGCC
Dld	CAGAGCTGGAGTCGTGTGTA	TCACGTCAGCCTCAATTGGTT
Pdha1	AGATGCTTGCCGCTGTATCC	GCCGATGAAGGTCACATTTCTTAAT
Dlat	ACAAGGCCACCATAGGCTTT	AGAGGAACATCCCTTGTGCC
Pdhb	AGTAGAGGACACGGGCAAGA	ACGAACTGTCAACTGCACCG
Nlrp3	CAAGGCTGCTATCTGGAGGAA	TGCAACGGACACTCGTCATC
IL-1β	TGCCACCTTTTGACAGTGATG	ATGTGCTGCTGCGAGATTTG
Casp1	CTATGGACAAGGCACGGGAC	TCAGCTGATGGAGCTGATTGA
Pink1	GAGGAGCAGACTCCCAGTTC	AGGGACAGCCATCTGAGTCC
Prkn	GCACACCCAACCTCAGACAA	GATGACAGAGGAAGATGACTGAC
Map1lc3b	GAGGGGACCCTAACCCCATA	TCGCTCTATAATCACCCGCC
Sqstm1	ACTGCTCAGGAGGAGACGAT	CCGGGGATCAGCCTCTGTAG
Map1lc3a	CGCTACAAGGGTGAGAAGCA	GCGGCGCCGGATGAT
Lamp1	GCCCTGGAATTGCAGTTTGG	TGCTGAATGTGGGCACTAGG
Lamp2	CAAAAGGACAGTATTCTACAGCTCA	TGATGGCGCTTGAGACCAAT
Ctsb	ATTCACACCAATGGCCGAGT	TAGCCACCATTACAGCCGTC

Bnip3	AAATTAAAGGGTGCGTGCGG	CAAAGTGGGGTTCGTGGGTA
Fundc1	CGTCCTTTAAAGTGGTGGGG	TCGCTTTCATAGTCTTGGGG
