# Supplementary figures and images for: Copper Chelation Therapy Attenuates Periodontitis Inflammation through the Cuproptosis/Autophagy/Lysosome Axis
Source: Int J Mol Sci. 2024 May 28;25(11):5890. doi: 10.3390/ijms25115890 (PMC11172687; doi:10.3390/ijms25115890)

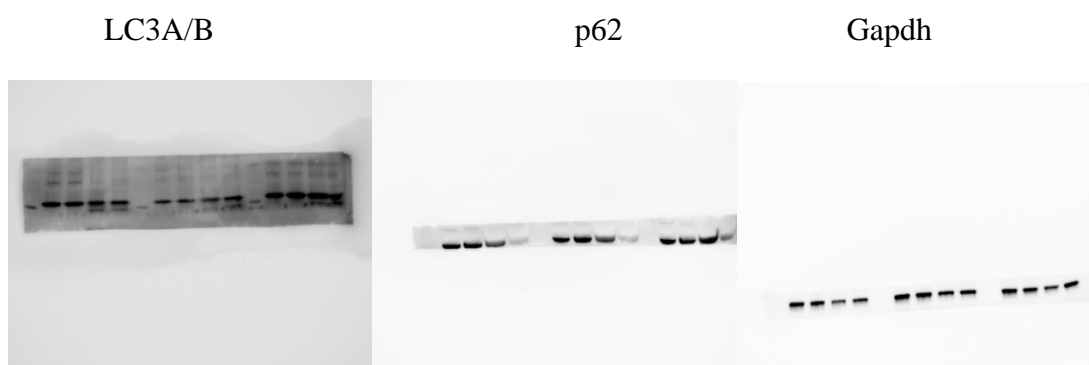

Original Western blot image of Figure 3D

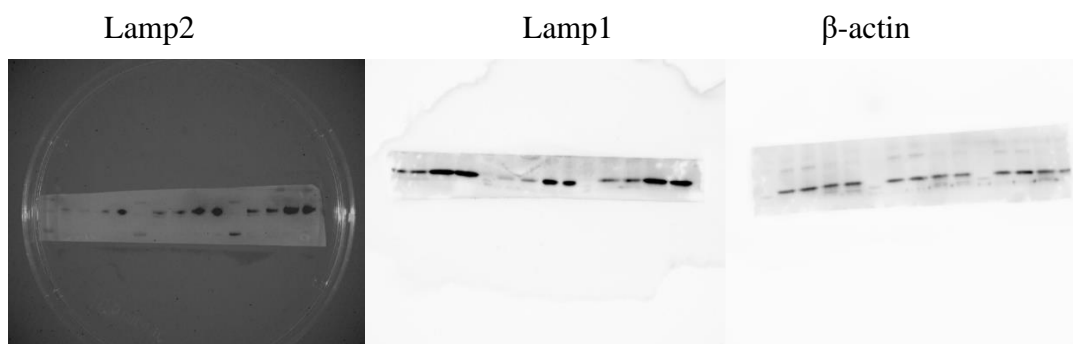

Original Western blot image of Figure 4C

Supplement: Supplementary file 1 [file ijms-25-05890-s001.zip › ijms-2971089-supplementary/Original Western blot image.pdf]
